# Leveraging Deep Learning and Generative AI for Predicting Rheological Properties and Material Compositions of 3D Printed Polyacrylamide Hydrogels

**DOI:** 10.3390/gels10100660

**Published:** 2024-10-15

**Authors:** Sakib Mohammad, Rafee Akand, Kaden M. Cook, Sabrina Nilufar, Farhan Chowdhury

**Affiliations:** 1 School of Electrical, Computer, and Biomedical Engineering, Southern Illinois University Carbondale, Carbondale, IL 62901, USA; sakib.mohammad@siu.edu; 2 School of Mechanical, Aerospace, and Materials Engineering, Southern Illinois University Carbondale, Carbondale, IL 62901, USA; rafee.akand@siu.edu (R.A.); kaden.cook@siu.edu (K.M.C.)

**Keywords:** deep learning, generative AI, 3D printing, polyacrylamide, rheology

## Abstract

Artificial intelligence (AI) has the ability to predict rheological properties and constituent composition of 3D-printed materials with appropriately trained models. However, these models are not currently available for use. In this work, we trained deep learning (DL) models to (1) predict the rheological properties, such as the storage (G’) and loss (G”) moduli, of 3D-printed polyacrylamide (PAA) substrates, and (2) predict the composition of materials and associated 3D printing parameters for a desired pair of G’ and G”. We employed a multilayer perceptron (MLP) and successfully predicted G’ and G” from seven gel constituent parameters in a multivariate regression process. We used a grid-search algorithm along with 10-fold cross validation to tune the hyperparameters of the MLP, and found the R^2^ value to be 0.89. Next, we adopted two generative DL models named variational autoencoder (VAE) and conditional variational autoencoder (CVAE) to learn data patterns and generate constituent compositions. With these generative models, we produced synthetic data with the same statistical distribution as the real data of actual hydrogel fabrication, which was then validated using Student’s *t*-test and an autoencoder (AE) anomaly detector. We found that none of the seven generated gel constituents were significantly different from the real data. Our trained DL models were successful in mapping the input–output relationship for the 3D-printed hydrogel substrates, which can predict multiple variables from a handful of input variables and vice versa.

## 1. Introduction

Traditionally made PAA hydrogels show linear elastic properties and have been widely used in mechanobiology studies [1]. Recently, we have demonstrated that 3D-printed PAA substrates show viscoelastic properties similar to living tissues [2], which opens exciting avenues for future explorations of mechanobiology studies. Using 3D printing techniques, the hydrogel properties can be tuned to get the desired mechanical and rheological properties that can be very beneficial to many investigations. However, 3D printing has several parameters, such as acrylamide concentration, bis-acrylamide concentration, photo-initiator concentration, layer height, and layer exposure time, to name a few. Each of these parameters has a profound impact on the mechanical and rheological properties of the substrate. Experimentation with each of the parameters and determining the corresponding outcome can be technically challenging, expensive, and time-consuming. This is where DL models can contribute by eliminating the need for experimental validation to determine the effect of each of the parameters.

DL is a subset of AI (or machine learning) that involves human brain-like architectures to mimic how our brain learns patterns [3]. It has achieved, and in some cases surpassed, human-level accuracy in complex tasks such as pattern recognition, computer vision, and natural language processing [4]. For finding patterns in high dimensional data, DL acts as an immensely powerful tool. Herein, we utilized an MLP, which is a DL model to predict rheological properties, namely G’ and G”, of 3D-printed PAA hydrogel substrates. Next, to predict gel constituents and printing parameters for a specified G’ and G”, we employed two generative models, namely VAE [5] and CVAE [6], to generate multiple combinations of gel constituents from a single value of G’ and G”. We validated our methodology for generating synthetic data with Student’s *t*-test and an AE [7] anomaly detector. With these models, the process of 3D printing of PAA hydrogel substrates will become more convenient and readily accessible.

## 2. Results and Discussion

### 2.1. The Overall Workflow Is Divided into Four Stages

**Figure 1:**
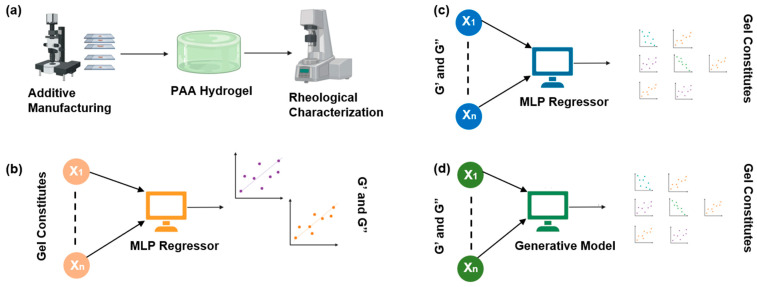
The workflow of our experiment is shown here. (**a**) Shows the method of data generation for AI model training. We 3D-printed hydrogel substrates using different material compositions, and printing parameters, and tested them to find their rheological properties, namely G’ and G”, at different frequencies. (**b**) An MLP regressor was used to predict G’ and G” from the hydrogel material constituents. (**c**) We predicted the hydrogel constituents from G’ and G” with an MLP. (**d**) Finally, we utilized VAE and CVAE to generate hydrogel material constituents that matched the original data.

The first stage involved working in the wet lab, where the PAA hydrogel substrates were manufactured with variations in the gel constituents and printing parameters (Figure 1a). The printing parameters include acrylamide concentration, bis-acrylamide concentration, photo-initiator concentration, layer height, bottom layer exposure time, and remaining layer exposure time. Next, we characterized their rheological properties, namely G’ and G”, at different frequencies of oscillation of the rheometer. We performed several replicates of experiments so that we could train our DL models reliably and without any underfitting or overfitting [8].

Our second stage of experiment (the first AI-based experiment) involved predicting the G’ and G” values from the hydrogel constituents and printing parameters. Using a multivariate MLP regressor [9], we predicted the output variables (G’ and G”) from the input variables (material constituents and printing parameters) (Figure 1b). But before proceeding with the training, we plotted the dataset and explored any correlation or pattern that may exist. Next, we trained the MLP with appropriate tuning of the hyperparameters [10] to predict G’ and G” reliably.

In the third stage of the experiment (second AI experiment), we predicted the hydrogel material parameters based on a pair of G’ and G” for a single combination of material constituents and printing parameters (Figure 1c) using an MLP. This would allow us to design and fabricate any hydrogels with desired rheological properties. However, this experiment did not pan out as expected; hence, we decided to utilize generative models in our experiment.

In the fourth stage (third and final AI experiment) of our experiment, we generated the gel constituents and printing parameters using generative models (Figure 1d). After testing several generative models, we decided to use a VAE and a CVAE. They reliably generated multiple combinations of gel constituents for a single G’ and G” value pair. Figure 1 shows the different stages of the workflow in a unified scheme.

### 2.2. Determining the Contribution of Each Parameter for Predicting G’ and G”

Supplementary Figure S1 shows the statistical distribution of our entire dataset, including the number of unique values in each column, column mean, median, standard deviation, and lower and upper quartiles. Supplementary Figure S2 shows the pair plots between every variable in our dataset. These values can later be compared to the synthetic data produced by the generative models to ensure that both have similar distributions. In Figure S3a, we utilized Pearson’s correlation matrix [11] to visualize the positive and negative correlations between all variables. We see that G’ and G” are positively correlated with both acrylamide and bis-acrylamide, and negatively correlated with layer height (Supplementary Figure S3b,c, respectively). Hence, the modulation of these parameters during the 3D printing of hydrogels highly affects the rheological properties.

**Figure 2:**
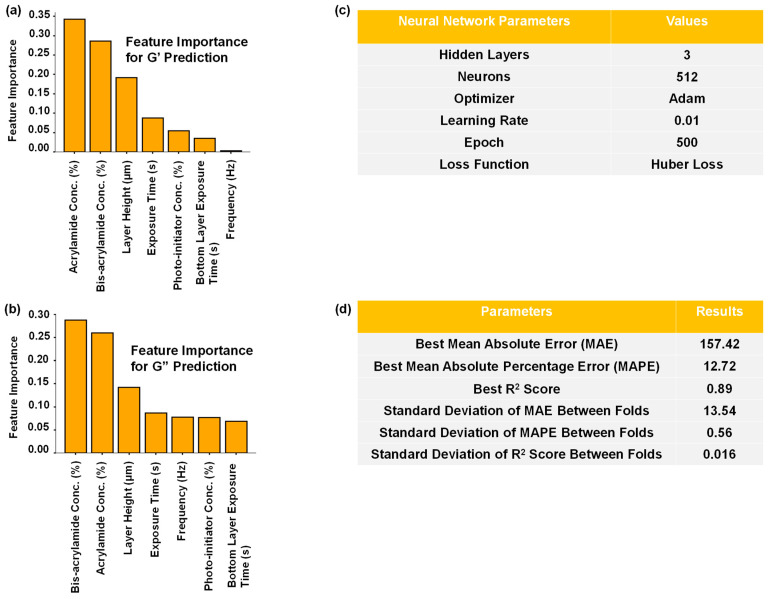
Results of the data analysis and G’ and G” prediction from hydrogel materials and printing parameters are presented. (**a**,**b**) show the feature importance of different variables for predicting G’ and G”, respectively, obtained from an XGBoost regressor. The feature importance of all variables adds up to 1. (**c**) Presents the hyperparameters of the MLP for the best result metrics. (**d**) Shows the results of the tuned MLP.

To understand the feature importance for predicting a variable, we used an XGBoost regressor [12], and the result is summarized in Figure 2a,b. It is to be noted that, while the correlation matrix refers to the actual relations among variables during fabrication, the feature importance indicates the features that the machine learning (ML) algorithm finds impactful for predicting a particular variable during training.

Finally, we have the best parameters for the tuned MLP in Figure 2c, which were obtained using a grid-search algorithm with a 10-fold cross-validation [13,14]. We found that the Huber Loss function [15], which is a combination of Mean Squared Error (MSE) and Mean Absolute Error (MAE) losses, worked the best for our model. The MSE loss did not work well for our study and led to exploding gradients and subsequent instability during training [16]. By adopting Huber Loss as a loss function, we obtained good results in every performance metric as shown in Figure 2d. Since we are performing a multivariate regression analysis, we used the Mean Absolute Percentage Error (MAPE) in addition to the MAE, to address the different scales of the output parameters. The high R^2^ score indicates that our model was successful in mapping the outputs from the inputs. As we employed a 10-fold cross-validation, it is important to make sure that we do not have high deviations between the folds in the performance metrics, which would indicate a lack of generalization for our MLP. From the results, we see that the deviation is very low, which means that our model is likely to perform well on unseen data.

### 2.3. Predicting the Hydrogel Constituents Using an MLP for a Specific Pair of G’ and G”

**Figure 3:**
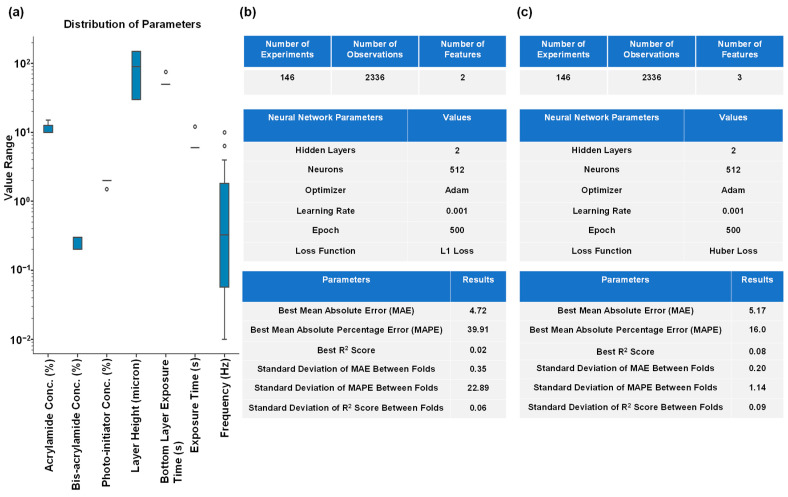
Results of prediction of the gel components from G’ and G” values using an MLP are delineated. (**a**) Shows the boxplots of the variables we predicted. (**b**) Shows the best hyperparameters and results for predicting the hydrogel materials from only G’ and G”. (**c**) Shows the optimum hyperparameters and results for predicting the gel parameters from G’, G” and frequency.

Our second AI experiment was to predict the gel composition for a paired G’ and G” combination. We predicted the gel composition from G’ and G” using an MLP in a similar fashion to our first AI experiment. However, it turned out to be challenging to predict seven output variables (the gel constituents) from only two input variables (G’ and G”). Therefore, we included frequency as an additional input variable. We presented the correlation among different features along with their respective statistical significance in Supplementary Figure S4, and the feature importance of the input variables (G’, G”, and frequency) for predicting each of the gel constituents in Supplementary Figure S5. Figure 3a displays the distribution of the predicting variables, which varies by several decades, and can make predicting and interpreting the output variables and results quite difficult. Although we anticipated these challenges, we proceeded with the training of the MLP, and Figure 3b,c shows the best hyperparameters for training the MLP and its results. From Figure 3b, we observed that while using only two variables (G’ and G”) as input parameters, the MLP did not perform well in predicting the hydrogel printing parameters. Therefore, we included frequency of oscillation during rheological measurements as an additional input variable. As a result, MAPE was improved, but not MAE or R^2^ scores (Figure 3c). We concluded that regression with an MLP model might not be suitable for our purpose and thus opted to use generative models.

### 2.4. Using Generative Models to Produce Multiple Gel Constituent Compositions for Paired G’ and G” Values

**Figure 4:**
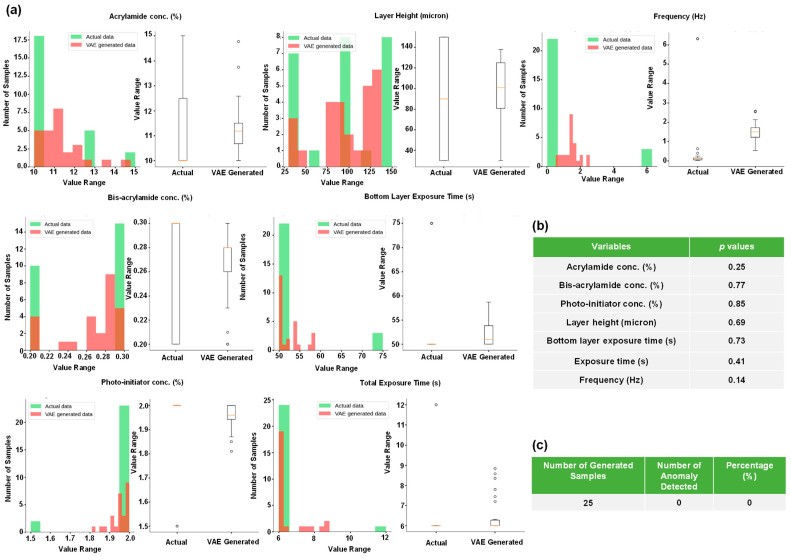
Similarities and distributions of the actual and synthetic data samples generated by the VAE are presented. (**a**) Shows histograms and boxplots of actual and VAE-generated synthetic data. (**b**) Shows the statistical similarities between actual and VAE-generated synthetic data based on Student’s *t*-test. (**c**) Shows the results of anomaly detection by the AE for the VAE-generated data when compared against actual data.

In this section, we produced the gel constituents from only G’ and G” using generative models. We leveraged two classes of AE, namely the VAE and the CVAE. Both VAEs were successful in producing multiple gel constituents for a paired G’ and G”. The use of VAE and CVAE were particularly advantageous over MLP as they can generate multiple sets of gel constituents from a single pair of G’ and G”, as opposed to a single set of values predicted by an MLP. Next, we verified our generated data in two ways. First, we utilized the Student’s *t*-test to determine if the generated and the real data are statistically similar. Second, we used an AE anomaly detector that learned the pattern of the real values and compared those against the generated values. The AE anomaly detector flags a generated sample as an anomaly if it is significantly distinct compared to the real values. Figure 4a shows the histograms and boxplots for each constituent of the hydrogel for both the real and generated data by the VAE. We observe that the histograms, for both synthetic and real data, overlap for acrylamide concentration, bis-acrylamide concentration, photo-initiator concentration, layer height, bottom layer exposure time, subsequent layer exposure time, and frequency. Figure 4b shows the *p*-values of each distribution, as determined by Student’s *t*-test, which indicates that the real and generated values are not statistically different. From Figure 4c, we also note that the anomaly detector found 0 aberration among the 25 sample data points generated by the VAE.

**Figure 5:**
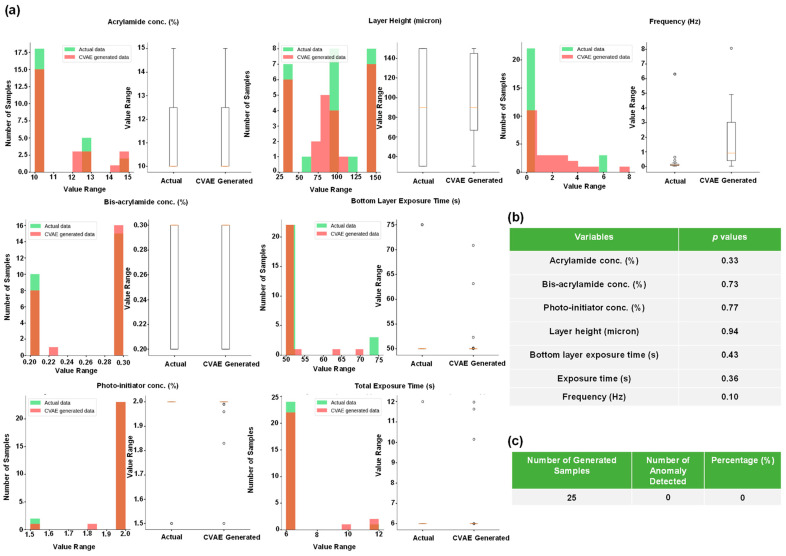
The distributions of the actual and synthetic data generated by the CVAE of the gel constituents are presented here. (**a**) Indicates boxplots and histograms of actual and synthetic data. (**b**) Shows the statistical similarities between the real and generated variables based on Student’s *t*-test. (**c**) Shows the results of the AE for the CVAE-generated data.

CVAE allows for setting bounds on data generation and provides more control over the generated data [17]. The results of CVAE data generation are displayed in Figure 5. In Figure 5a, we observe that the CVAE data histograms overlap better with the real data when compared with VAE-generated data. In addition, the AE did not find any anomaly in the generated samples (Figure 5c). Overall, CVAE captured the distribution of the real data better than the VAE.

The current work provides an excellent prediction capability for the 3D printing of PAA hydrogels. Our trained models can predict the rheological properties (G’ and G”) of PAA hydrogels from a combination of PAA gel constituents and printing parameters. In addition, we also demonstrated that we can generate the PAA gel constituents and printing parameters for paired G’ and G” values. However, there are caveats to our experiments as with any ML/AI applications. Our trained models performed well with high precision based on the modest dataset that we generated. In principle, if we had a larger dataset, the variations in input and output parameters could be captured even better. With more data, we could make the model even more accurate and generalized.

We selected seven input parameters to design our DL studies to provide two key rheological properties, G’ and G”. These parameters include resin composition (acrylamide, bis-acrylamide, photo-initiator concentrations), 3D printing parameters (layer height, bottom layer exposure time, exposure time), and frequency of oscillation of the rheometer. The composition of the resin plays a critical role in determining the mechanical properties of the hydrogel. Specifically, the ratios of the monomer, crosslinker, and photo-initiator in the resin govern the degree of polymerization that occurs in each layer during curing. The monomer provides the building blocks for the polymer network, while the crosslinker controls the degree of network connectivity, affecting viscoelasticity. The photo-initiator initiates polymerization when exposed to ultraviolet (UV) light, and its concentration influences the reaction kinetics. A careful balance of these components is necessary to optimize the gel’s mechanical properties. In stereolithography (SLA) 3D printing, structures are built by layer-by-layer polymerization. The thickness of each layer plays a significant role in determining how much UV light penetrates the gel, thereby affecting the degree of polymerization within that layer. The exposure time to UV light for each individual layer is also a critical factor. In typical SLA printing, the first few layers are exposed to UV light for a longer duration to ensure a robust foundation for printing. This extended exposure results in higher polymerization and thus increased mechanical stability in the foundational layers. However, for the remaining layers, the duration of UV exposure continues to dictate the degree of polymerization, directly impacting the mechanical properties of the gel, such as G’ and G”. By controlling parameters like layer thickness and UV exposure time, we can fine-tune the properties of the hydrogel, making it suitable for specific biological or mechanical applications. Finally, we selected frequency of oscillation as an input parameter for predicting G’ and G” because viscoelasticity is a time-dependent property, which describes the materials’ elastic (solid-like) and dissipative (fluid-like) behaviors when subjected to deformation. The frequency sweep test is essential for characterizing viscoelastic materials because it provides detailed insights into how the material behaves across different timescales.

The generative models (VAE and CVAE), as opposed to the MLP, capture data as a distribution, while MLP models capture and predict data as discrete points for regression operation. This is potentially a reason why the MLP failed to perform well in predicting the hydrogel constituents, which have a varying value range, in addition to the challenge of predicting a high number of variables from a handful of input variables. In addition to VAE and CVAE, we worked with two additional generative models, namely the Generative Adversarial Network (GAN) [18] and the Conditional Generative Adversarial Network (cGAN) [19]. However, training these models led to some instability, as evident in Supplementary Figure S6a,b. We expected the generator loss to be decreasing, while the discriminator loss should be increasing, which is clearly not the case [20,21]. Since VAE and CVAE are more pertinent to our data type and had already generated reliable synthetic data, we limited our effort to optimize the training of GAN and cGAN.

In a recent work, Verheyen and co-workers incorporated experimental data and AI-based methods into 3D printing processes to produce controlled structures, rheological properties, and injectability profiles for granular matrices of alginate bio-blocks [22]. Similar to their approach, we also combined data-driven AI methods for predicting rheological properties for the 3D printing of PAA hydrogels. However, in our study, we mainly focused on 1) predicting the rheological properties (G’ and G”) of PAA hydrogels from a combination of PAA gel materials and printing parameters, and 2) generating the PAA gel constituents for paired G’ and G” rheological properties. PAA hydrogels are more heavily studied and have garnered a lot of interest in the mechanobiology field, which makes our study relevant to a larger scientific community [23,24,25,26,27,28,29,30,31]. Our major contribution was to utilize generative models to produce multiple sets of gel constituent values from a single pair of G’ and G” that are statistically significant, whereas Verheyen et al. worked exclusively on predictive studies regarding the outcome of several 3D printing processes, without utilizing any generative models. By combining our methods, one can reliably predict the rheological properties (G’ and G”) of 3D-printed PAA hydrogels and generate reliable sets of gel constituents from a pair of G’ and G”.

## 3. Conclusions

We trained and validated deep learning models and leveraged AI technologies for reliable prediction of rheological properties and material compositions of PAA hydrogels. The experimental rheology data was produced in our laboratory with varying input parameters. Since the 3D printing process depends on these input variables, AI technology can be beneficial for defining the process parameters or conversely predicting rheological properties of the PAA hydrogel without the need for iterative experimentations. We used MLP to predict G’ and G” and VAE/ CVAE to generate material process parameters for 3D printing of PAA hydrogels. In summary, deep learning models and generative AI provide process parameters and material property insights without experimentation.

## 4. Materials and Methods

### 4.1. Fabrication of PAA Hydrogels by 3D Printing

Glass slides measuring 75 mm × 25 mm were cleaned with double-distilled water (ddH_2_O), followed by smearing of 200 µL of 97% 3-aminopropylme-thoxysilane (APTMS) (Sigma Aldrich, St. Louis, MO, USA; cat. # 281778-100ML) over the glass surface using a cotton swab for 15 min. The glass slides were rinsed with ddH_2_O and then submerged in 0.5% APTMS in ddH_2_O for 10 min while being stirred. Next, these slides were washed using ddH_2_O on an orbital shaker for 10 min. The slides were then placed inside a convection oven and baked at 160 °C for an hour. These were cooled and incubated with 500 µL of 0.5% grade-II glutaraldehyde (Sigma Aldrich; cat. # G6257-100ML) for 30 min. Activated slides were washed 2× with ddH_2_O for 15 min to remove excess glutaraldehyde.

Varying concentrations of acrylamide solution (Bio-Rad, Hercules, CA, USA; cat. # 1610146), bis-acrylamide solution (Bio-Rad, cat. # 1610142), and photo-initiator lithium phenyl-2,4,6-trimethylbenzoylphosphinate (Arkema Inc., King of Prussia, PA, USA; cat. # CPS Proprietary Initiator: TPO-Li) were physically mixed using a vortex mixer. As the photo-initiator was light-sensitive, the mixture was kept in an opaque container in a dark chamber.

We used an SLA-based 3D printing technique with a Phrozen Sonic Mini (Phrozen Tech Co., Ltd., Hsinchu City, Taiwan) printer to print the hydrogels. The freshly prepared photoreactive acrylamide and bis-acrylamide mixture was poured into the vat and the activated glass slides were placed on the platform using double-sided tapes. The SLA 3D printer we used has a bottom-up setup, where a 405 nm UV light is placed under the vat, and light is projected through the vat in the upward direction. The glass platform slide was lowered into the vat and a fixed preset gap was maintained between the glass platform and the bottom of the vat. This gap is also called layer height. Next, the UV light was projected on the photoreactive mixture between the glass platform and the bottom of the vat. Once a single layer is exposed for a given exposure time, a single layer is polymerized, and the printing base is raised by the amount of a single layer height. For the first few layers, the UV exposure time was kept longer (varying from 50–75 s) as they formed a stronger base of the print, and for the rest of the consecutive layers, the exposure time varied from 6–12 s. As 3D printing gives us greater control over the printing process, we identified and varied some of the key parameters during the printing process, such as the layer height, bottom layer exposure time, consecutive layer exposure time, and so on. As a result, we produced PAA hydrogels of varying mechanical properties. The composition of the mixture was also varied by using different concentrations of acrylamide, bis-acrylamide, and photo-initiator. After the print was complete, the hydrogels were submerged in phosphate buffer saline (PBS) (Thermo Fisher Scientific, Waltham, MA, USA; cat. # 10010023) for 24 h before taking rheological measurements.

#### 4.1.1. Measuring Rheological Properties of 3D-Printed PAA Hydrogels

To investigate the effect of the varying key parameters on the viscoelastic properties of the 3D-printed hydrogels, small-amplitude oscillatory shear (SAOS) rheology was used. A HAAKE MARS 60 (Thermo Fisher Scientific, Waltham, MA, USA) rotational rheometer was used for all rheological measurements. All measurements were obtained at room temperature (25 °C). Hydrogels were printed as 21 mm circular disks with 2 mm height and were allowed to swell up completely for 24 h before any rheological measurements. A pair of serrated plates of 20 mm diameter was used, and the gap between the parallel plates was kept between 1.6 and 1.7 mm so that the normal force exerted on the samples was lower than 1 N. The excess gels were trimmed off using a surgical blade after lowering the plates to their measuring positions. Deformation amplitude sweeps were performed to assess the upper limit of the linear viscoelastic region (LVR) for each type of sample, based on a 5% decrease from the storage modulus G’ plateau at a frequency of 1 Hz and strain values starting from 0.001 Hz. To measure G’ and G” of the samples over several decades of frequency, sweeps were performed with frequencies ranging from 0.01 Hz to 10 Hz with a 10 Pa shear force, which was within the LVR range for all samples. A total of 5 measurements were taken in each decade of frequencies.

#### 4.1.2. Rheology Data Collection and Processing

G’ and G” corresponding to each frequency tested were collected, and the raw data was exported to an Excel file. Since rheological measurements are very sensitive to even small material deviations or impurities within the sample, three to six samples were printed and tested for each condition.

### 4.2. DL Experiments for Predicting Material Properties and Printing Parameters

After collection, data was stored in an Excel file for DL model training. First, we performed an exploratory data analysis (EDA) to visualize data patterns and establish relationships among the variables. After performing EDA, we gained important insights about the data and built our models accordingly. We divided the AI experiments into three parts. For the first part, we aimed to predict G’ and G” from the hydrogel constituents and frequencies of oscillation at which the moduli were measured. It is a multivariate regression problem, and we decided to use MLP because of its simple architecture. In addition, we employed an XGBoost model to find out the feature importance for predicting a single output variable. To train our MLP, we used a grid-search-based approach for hyperparameter tuning over k-folds (in our case, it was a 10-fold cross-validation). Employing a k-fold cross-validation method increased the robustness of our model over the full dataset, which in turn helped to improve the generalization. We used dropout layers [32] in the MLP to further prevent overfitting. Finally, we calculated the R^2^ score, MAE, and MAPE for each fold and averaged them over 10 folds, which was reported in addition to standard deviations of these scores between the folds. As we had two output variables to predict, the reported MAE was the average of these two variables. However, as the output variables have different scales, as mentioned before, we also reported the MAPE values. In the second part of our experiment, we predicted the gel constituents from the values of G’ and G” using an MLP regressor. The setup of this experiment was similar to the first one. Here, instead of putting seven variables to the input side and receiving two variables on the output side, we performed the reverse experiment. In other words, we sought to predict the composition of the gels from only G’ and G” values. The training setup was identical in terms of grid search and k-fold cross-validation methods. However, the results of this experiment were not satisfactory. As a result, we decided to put the frequency of oscillation as an additional input variable and predicted six output variables from three input variables. This improved the results of the MLP a bit but still did not meet our expectations. Finally, we opted for generative models (VAE and CVAE) to improve the structure of our experiment. Both VAE and CVAE have an encoder–decoder style architecture. VAE learns the data patterns by encoding the information in a low-dimensional latent space taking the shape of a probability distribution (generally normal distribution). Then, the model decodes points from the latent space to generate new synthetic data points. This ensures that the data maintain similarities with the original data. The CVAE functions similarly to VAE; however, it adds additional conditional variables in addition to the input variables, allowing control over certain aspects of the data generated. This makes CVAE particularly useful in generating data with specific properties, offering more control over the data generated, as opposed to VAE’s purely unsupervised generation. For training both models, a combination of MSE and Kullback–Leiber Divergence (KLD) [33] loss function was utilized. The KLD portion of the loss function ensures that the latent space follows a normal distribution, while the MSE portion makes sure that reconstructed data from the decoder closely resembles the input data. The final task was to verify the data we received from the generative models. We performed two additional experiments for this purpose. First, we utilized an AE anomaly detector. It deconstructed the real data and learned its distribution, and then compared it with the generated data. If the distribution of the generated data fell outside the distribution of the real data, then the AE would flag it as an anomaly. Secondly, we used the Student’s *t*-test to determine if the real and generated data distributions were significantly different. Utilizing these two verification methods, we validated the quality of the generated data. We should mention that all the data were normalized before going to the deep learning models as normalization is essential for training those models. The loss functions we used to train the DL models throughout this study are presented below.

$$MSE=\frac{1}{N}\sum_{i=1}^{N}{\left(y_{i}-{\hat{y}}_{i}\right)}^{2}$$

$$MAE=\frac{1}{N}\sum_{j=1}^{N}\left|y_{j}-{\hat{y}}_{j}\right|$$

$$HuberLoss=\left\{\begin{array}{c} \frac{1}{2}{\left(y_{i}-{\hat{y}}_{i}\right)}^{2}\;for\;\left|y_{i}-{\hat{y}}_{i}\right|\leq \partial \\ \partial \left|y_{i}-{\hat{y}}_{i}\right|\;-\;\frac{1}{2}\partial^{2}\;otherwise \end{array}\right.$$

$$VAE\;Loss=MSE\left(y,\hat{y}\right)+0.001\times KLD(q_{\phi }(z|x)||p(z))$$

For AI experiments, we used Python programming language. The libraries we utilized were the following: NumPy [34], Pandas [35], Scikit-learn [36] for processing the dataset, SciPy [37] to perform statistical tests, XGBoost to find out feature importance, PyTorch [38] for the DL models, and finally, Matplotlib [39] and Seaborn [40] to visualize the data and results. We trained the generative models on Google Colab and the MLP models on a local system.

## Data Availability

The data and codes are available at: https://github.com/sakibmohammad/hydrogel_rheology_project.git (accessed on 14 October 2024).

## Supplementary Materials

The following supporting information can be downloaded at: https://www.mdpi.com/article/10.3390/gels10100660/s1, Figure S1: Statistical distribution of the dataset; Figure S2. Pair plots of all variables; Figure S3. Correlation of different features for the regression operation; Figure S4. Correlation of variables for hydrogel constituents’ prediction; Figure S5. Feature importance for hydrogel constituents’ prediction; Figure S6. Training losses for the GAN models.
