# Supplementary material for: Leveraging Deep Learning and Generative AI for Predicting Rheological Properties and Material Compositions of 3D Printed Polyacrylamide Hydrogels
Source: Gels. 2024 Oct 15;10(10):660. doi: 10.3390/gels10100660 (PMC11507415; doi:10.3390/gels10100660)
Supplement: Supplementary file 1 [file gels-10-00660-s001.zip › gels-3251382-supplementary.pdf]

Variables	Unique Values	Mean	Median	Variance	Standard Deviation	Min Value	25% Value	50% Values	75% Value	Max Value
Acrylamide conc. (%)	3	11.58	10.00	4.03	2	10.00	10.00	10.00	12.50	15.00
Bis-acrylamide conc. (%)	2	0.27	0.30	0.002	0.05	0.20	0.20	0.30	0.30	0.30
Photo-initiator conc. (%)	2	1.95	2.00	0.023	0.15	1.50	2.00	2.00	2.00	2.00
Layer height (micron)	5	93.08	90.00	2253.74	47.47	30.00	30.00	90.00	150.00	150.00
Bottom layer exposure time (s)	2	52.40	50.00	54.21	7.36	50.00	50.00	50.00	50.00	75.00
Exposure time (s)	2	6.58	6.00	3.12	1.77	6.00	6.00	6.00	6.00	12.00
Frequency (Hz)	29	1.69	0.32	7.52	2.74	0.01	0.06	0.32	1.82	10.0
Storage Modulus (Pa)	2329	3365.00	1631.75	6929652	2632.42	252.07	1679.13	2631.75	4006.16	13550.49
Loss Modulus (Pa)	2325	229.00	199.12	20680.55	143.81	44.86	140.47	199.12	275.73	1298.21

Supplementary Figure S1. Statistical distribution of the dataset.

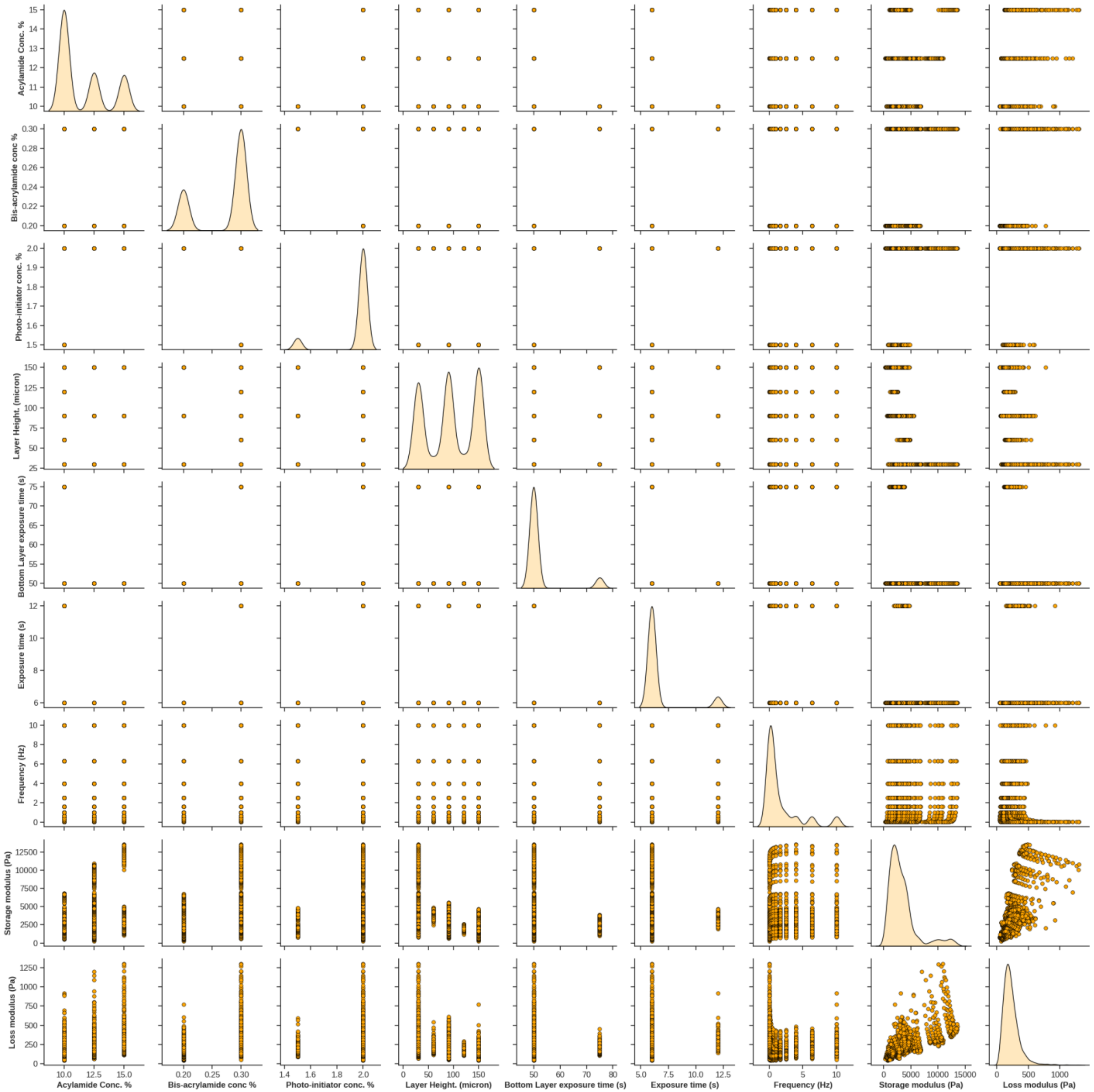

Supplementary Figure S2. Pair plots of all variables.

(a) Correlation Matrix of all Variables

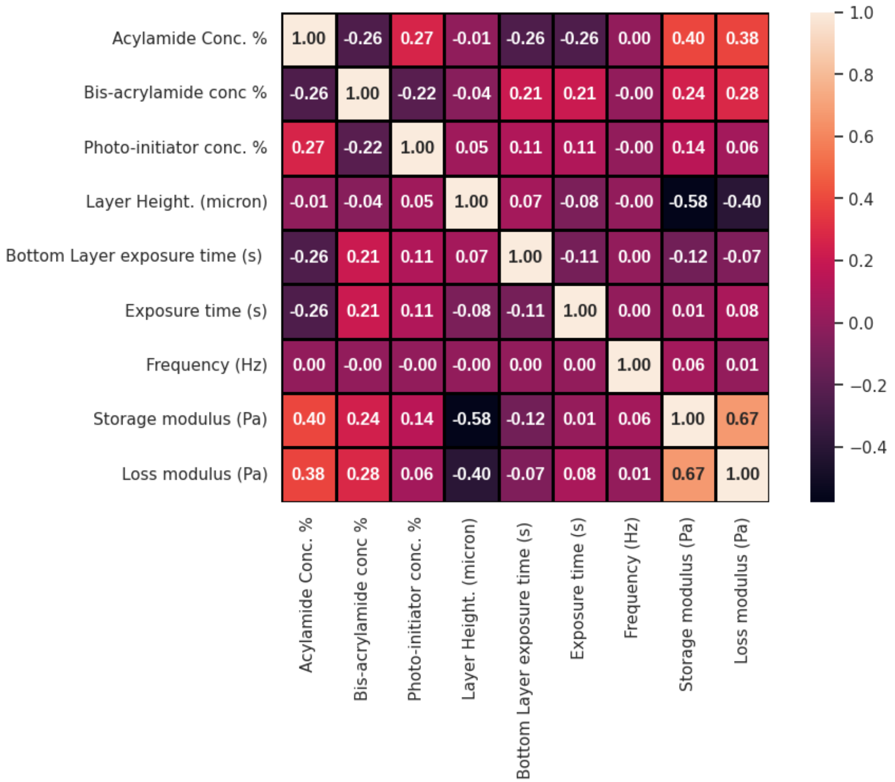

(b) Correlation of Variables for Storage Modulus

Variables	Pearson's Correlation	Significance (p<0.05)
Acrylamide conc. (%)	0.40	Yes
Bis-acrylamide conc. (%)	0.24	Yes
Photo-initiator conc. (%)	0.15	Yes
Layer height (micron)	-0.58	Yes
Bottom layer exposure time (s)	-0.12	Yes
Exposure time (s)	0.009	No
Frequency (Hz)	0.06	Yes

(c) Correlation of Variables for Loss Modulus

Variables	Pearson's Correlation	Significance (p<0.05)
Acrylamide conc. (%)	0.38	Yes
Bis-acrylamide conc. (%)	0.28	Yes
Photo-initiator conc. (%)	0.06	Yes
Layer height (micron)	-0.40	Yes
Bottom layer exposure time (s)	-0.07	Yes
Exposure time (s)	0.08	Yes
Frequency (Hz)	0.006	No

Supplementary Figure S3. Correlation of different features for the regression operation for **(a)** all variables, **(b)** predicting storage modulus (G'), and **(c)** predicting loss modulus (G").

Correlation of Variables for Acrylamide Conc.

Variables	Pearson's Correlation	Significance ($p<0.05$)
Frequency (Hz)	2.95e-07	No
Storage Modulus (Pa)	0.40	Yes
Loss Modulus (Pa)	0.38	Yes

Correlation of Variables for Bis-acrylamide Conc.

Variables	Pearson's Correlation	Significance ($p<0.05$)
Frequency (Hz)	-2.39e-07	No
Storage Modulus (Pa)	0.24	Yes
Loss Modulus (Pa)	0.28	Yes

Correlation of Variables for Photo-initiator Conc.

Variables	Pearson's Correlation	Significance ($p<0.05$)
Frequency (Hz)	-1.27e-07	No
Storage Modulus (Pa)	0.14	Yes
Loss Modulus (Pa)	0.06	Yes

Correlation of Variables for Layer Height

Variables	Pearson's Correlation	Significance ($p<0.05$)
Frequency (Hz)	-7.06e-08	No
Storage Modulus (Pa)	-0.58	Yes
Loss Modulus (Pa)	-0.40	Yes

Correlation of Variables for Bottom Layer Exposure Time

Variables	Pearson's Correlation	Significance ($p<0.05$)
Frequency (Hz)	1.22e-07	No
Storage Modulus (Pa)	-0.12	Yes
Loss Modulus (Pa)	-0.07	Yes

Correlation of Variables for Exposure Time

Variables	Pearson's Correlation	Significance ($p<0.05$)
Frequency (Hz)	1.22e-07	No
Storage Modulus (Pa)	0.009	No
Loss Modulus (Pa)	0.08	Yes

Supplementary Figure S4. Correlation of variables for hydrogel constituents' prediction.

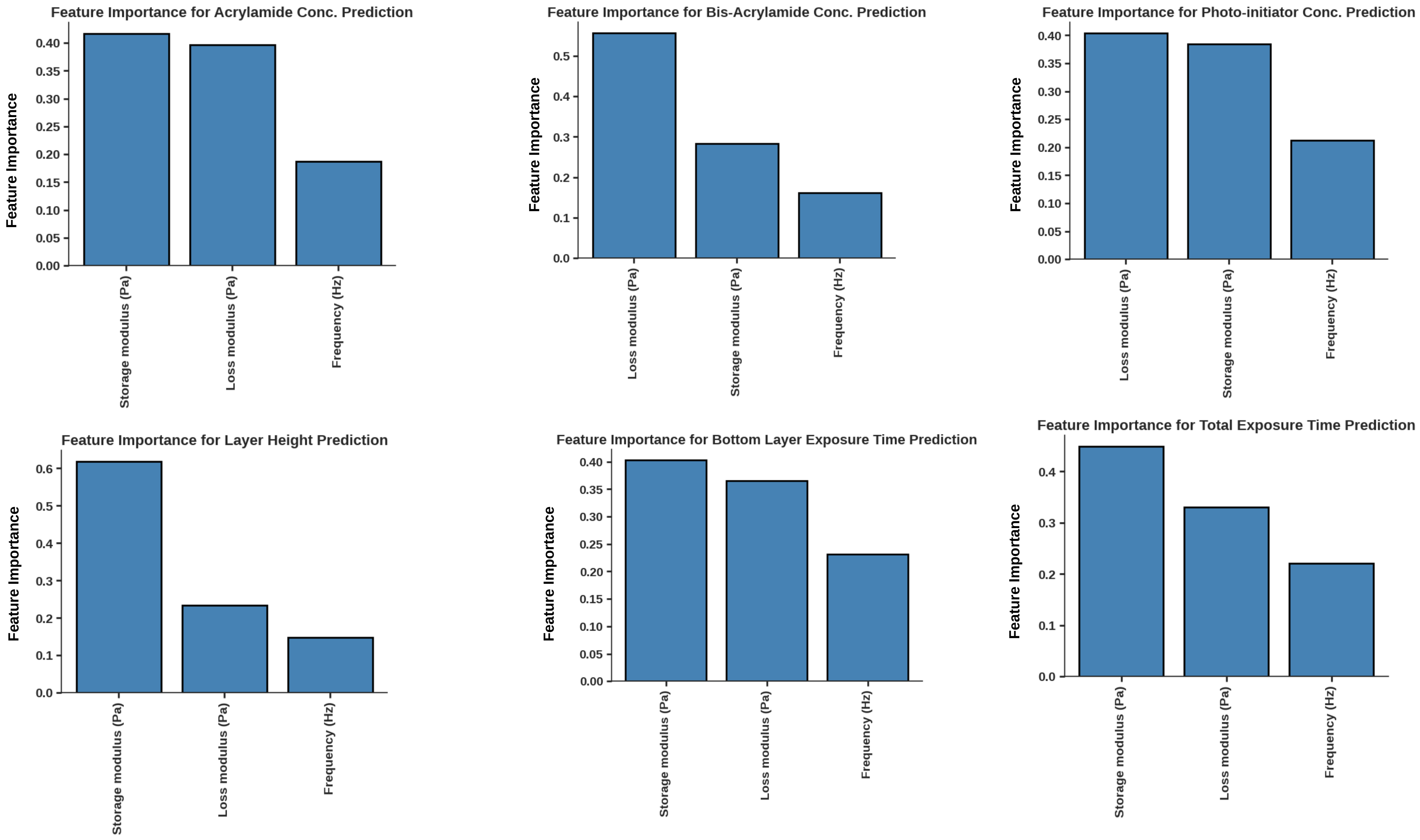

Supplementary Figure S5. Feature importance for hydrogel constituents' prediction.

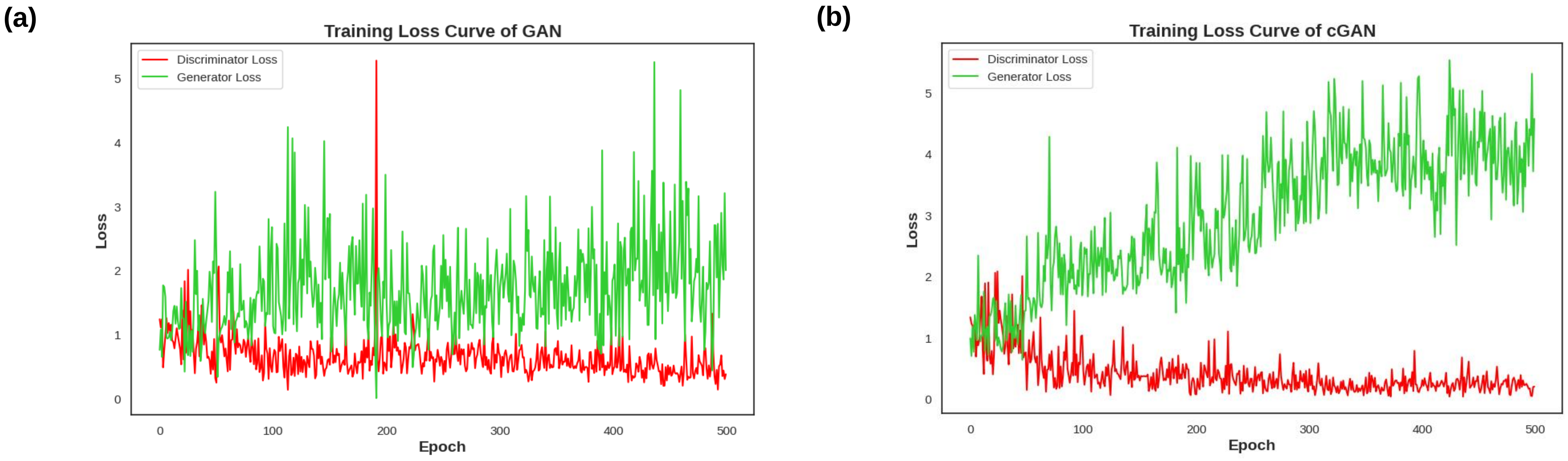

Supplementary Figure S6. Training losses for the GAN models. (a) GAN training loss. (b) cGAN training loss.
